# Noninvasive mechanical destruction of liver tissue and tissue decellularisation by pressure-modulated shockwave histotripsy

**DOI:** 10.3389/fimmu.2023.1150416

**Published:** 2023-05-16

**Authors:** Ki Joo Pahk, Jeongmin Heo, Chanmin Joung, Kisoo Pahk

**Affiliations:** ^1^ Department of Biomedical Engineering, Kyung Hee University, Yongin, Republic of Korea; ^2^ Center for Bionics, Biomedical Research Institute, Korea Institute of Science and Technology, Seoul, Republic of Korea; ^3^ Graduate School of Biomedical Sciences, University of Texas Southwestern Medical Center, Dallas, TX, United States; ^4^ Department of Nuclear Medicine, Korea University College of Medicine, Seoul, Republic of Korea

**Keywords:** high intensity focused ultrasound, boiling histotripsy, pressure-modulated shockwave histotripsy, cavitation, tumour destruction, immunotherapy, tissue decellularisation

## Abstract

**Introduction:**

Boiling histotripsy (BH) is a promising High Intensity Focused Ultrasound (HIFU) technique that can be used to mechanically fractionate solid tumours at the HIFU focus noninvasively, promoting tumour immunity. Because of the occurrence of shock scattering phenomenon during BH process, the treatment accuracy of BH is, however, somewhat limited. To induce more localised and selective tissue destruction, the concept of pressure modulation has recently been proposed in our previous *in vitro* tissue phantom study. The aim of the present study was therefore to investigate whether this newly developed histotripsy approach termed pressure-modulated shockwave histotripsy (PSH) can be used to induce localised mechanical tissue fractionation in *in vivo* animal model.

**Methods:**

In the present study, 8 Sprague Dawley rats underwent the PSH treatment and were sacrificed immediately after the exposure for morphological and histological analyses (paraffin embedded liver tissue sections were stained with H&E and MT). Partially exteriorised rat’s left lateral liver lobe *in vivo* was exposed to a 2.0 MHz HIFU transducer with peak positive (*P*_+_) and negative (*P*_-_) pressures of 89.1 MPa and –14.6 MPa, a pulse length of 5 to 34 ms, a pressure modulation time at 4 ms where *P*_+_ and *P*_-_ decreased to 29.9 MPa and – 9.6 MPa, a pulse repetition frequency of 1 Hz, a duty cycle of 1% and number of pulses of 1 to 20. Three lesions were produced on each animal. For comparison, the same exposure condition but no pressure modulation was also employed to create a number of lesions in the liver.

**Results and Discussion:**

Experimental results showed that a partial mechanical destruction of liver tissue in the form of an oval in the absence of thermal damage was clearly observed at the HIFU focus after the PSH exposure. With a single pulse length of 7 ms, a PSH lesion created in the liver was measured to be a length of 1.04 ± 0.04 mm and a width of 0.87 ± 0.21 mm which was 2.37 times in length (*p* = 0.027) and 1.35 times in width (*p* = 0.1295) smaller than a lesion produced by no pressure modulation approach (e.g., BH). It was also observed that the length of a PSH lesion gradually grew towards the opposite direction to the HIFU source along the axial direction with the PSH pulse length, eventually leading to the generation of an elongated lesion in the liver. In addition, our experimental results demonstrated the feasibility of inducing partial decellularisation effect where liver tissue was partially destructed with intact extracellular matrix (i.e., intact fibrillar collagen) with the shortest PSH pulse length. Taken together, these results suggest that PSH could be used to induce a highly localised tissue fractionation with a desired degree of mechanical damage from complete tissue fractionation to tissue decellularisation through controlling the dynamics of boiling bubbles without inducing the shock scattering effect.

## 1 Introduction

Cancer is the second major cause of death worldwide after cardiovascular diseases (1, 2). The estimated number of new cancer cases diagnosed in the United States is reported to be 1,918,030 and approximately 609,360 people will die from cancer in 2022 (3). Among many different types of cancers, liver cancer is the 6^th^ most common cancer and the 4^th^ leading cause of cancer-related death with 841,080 cases diagnosed in 2018 worldwide. Patients with early-stage liver cancer can be treated with surgical liver resection or percutaneous ablation through radiofrequency, microwave, cryoablation or trans-arterial radioembolization (4). Hepatectomy has been the mainstream treatment for early-stage liver cancer; however, the invasive surgical procedures can possibly lead to certain surgical complications such as bleeding, infection, blood clots and pneumonia. It has been reported that the most effective and widely used ablation therapies for treating liver cancer are radiofrequency and microwave ablations which both rely on a thermal ablative effect and require needle punctures (i.e., minimally invasive) (5). Because the heat sink effect significantly appears during these thermal-based treatments, it is difficult to precisely predict and control thermal ablation regions in real-time, thereby possibly increasing tumour recurrence rate (6–8). Ionising radiation therapy can also be employed as being a noninvasive clinical method; however, toxicities and a penumbra of nonlethal doses surrounding the targeted tissue are major concerns (5, 9).

High Intensity Focused Ultrasound (HIFU) is a non-invasive and non-ionising ultrasound technique which uses a focused ultrasound beam to treat cancer at the HIFU focus without disruption of surrounding tissue (10–12). In general, two different biological effects can be obtained by HIFU: thermal effect and mechanical effect. The production of heat at the HIFU focus can be used to thermally ablate solid tumours, whereas acoustic cavitation which can form as a result of mechanical effect of HIFU can lead to the mechanical destruction of cancer cells at the focus. The latter technique is known as boiling histotripsy (BH) (13). The overall shape of a HIFU thermal lesion is ellipsoid (10) whilst that of a BH lesion is tadpole like consisting of a head and a tail with the head closest to the HIFU transducer (13). In recent years, BH has gained more interest than HIFU thermal ablation in the fields of tumour ablation and immunotherapy. A number of previous studies have clearly demonstrated that BH can be used to mechanically fractionate different types of soft tissues (liver, kidney, brain, prostate, etc) and cancer cells (renal carcinoma, breast cancer, colorectal cancer, prostate cancer, etc) without causing any significant thermal damage (14–23) as well as can promote immunogenic cell death of cancer cells *via* TNF-induced necrosis signalling pathway (21). Since BH does not generate denatured tumour antigens at the treatment site whilst HIFU thermal ablation leads to protein denaturation, BH can release relatively more damage-associated molecular patterns (CRT, HSP 70, HMGB-1), pro-inflammatory cytokines (IFN-γ, IL-1α, IL-1β, IL-18) and chemokines (IL-8) than those released by HIFU thermal ablation, resulting in enhanced dendritic cells activation and generation of CD8^+^ T cells (21, 22, 24, 25). BH can therefore lead to stronger and longer lasting immune responses than those triggered by HIFU thermal ablation (22, 26).

In BH, nonlinear shocked waves with high acoustic peak pressure amplitudes produced at the HIFU focus in soft tissue can increase tissue temperature to boiling in a few milliseconds followed by the formation of a vapour bubble, which is known as shock wave heating effect (13, 27). Further interaction of incoming incident shockwaves with scattered acoustic waves by the boiling bubble can then induce a greater peak negative pressure field between the HIFU source and the bubble, leading to additional cavitation nucleation sites (28–31). When the peak negative pressure magnitude of this localised backscattered pressure field becomes greater than the intrinsic cavitation cloud threshold (e.g., – 28 MPa in most soft tissue), a cavitation cluster can appear which is known as shock scattering effect (15, 32). Mechanical shear stresses produced around boiling bubbles can tear off surrounding tissue, leading to the formation of a tail of a BH lesion and emission of micro-jetting and shockwaves resulting from inertial cavitation clouds enable tissue destruction, producing the head of a BH lesion (28–31). Along with these, atomisation and microfountain effects (33, 34) may further facilitate the process of mechanical destruction during BH exposure.

Whilst numerous *in vitro*, *ex vivo* and *in vivo* studies have shown the promises of using BH for treating tumours and promoting tumour immunity in the tumour microenvironment, the degree of control over mechanical damage to tissue by BH is, however, somewhat limited (32) because of the shock scattering effect, which is a stochastic phenomenon (30, 31) and depends upon the pressure magnitude of a backscattered acoustic field by a boiling bubble (30) and the location and number of pre-existing bubble nuclei within soft tissue (13, 35). To improve the treatment accuracy, a new histotripsy method termed pressure-modulated shockwave histotripsy (PSH) has recently been proposed and demonstrated in liver tissue phantom *in vitro* (32). In contrast to BH that uses pulsed shockwaves with a constant pressure amplitude, PSH employs a specifically designed pressure-modulated HIFU pulsing protocol where, within a single PSH pulse, shockwaves with high peak positive (*P*_1,+_) and negative pressure (*P*_1,-_) amplitudes at the HIFU focus are initially used to create a boiling bubble *via* localised shockwave heating and subsequent weakly distorted nonlinear or linear ultrasound waves with lower pressure amplitudes (*P*_2,+_ and *P*_2,-_) are applied to maintain the boiling bubble without inducing the shock scattering effect. The concept of PSH has been well demonstrated through observing PSH-induced bubble dynamics in liver tissue phantom with a high-speed camera (32); however, its biological effects on living tissue have not yet been studied. To investigate the characteristics of a freshly created PSH lesion in the liver, *in vivo* animal experiments are, therefore, conducted in the present study. A wide range of PSH exposure conditions was employed to examine potential changes of extent and degree of mechanical damage generated in animal’s liver morphologically and histologically.

## 2 Materials and methods

### 2.1 PSH experimental *in vivo* setup and exposure conditions

**Figure 1:**
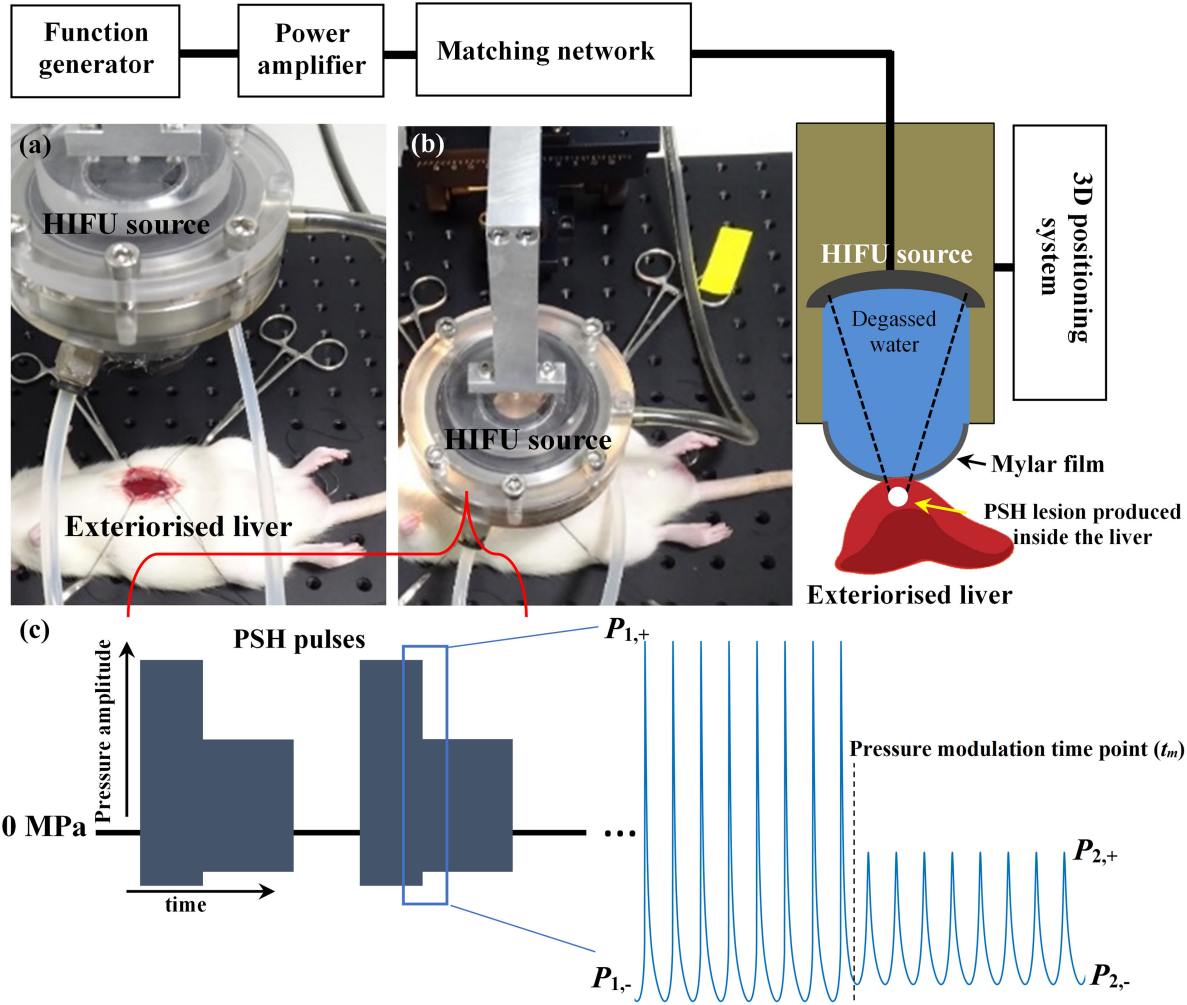
HIFU experimental setup used in the present study. Photographs of **(A)** exteriorised liver prior to HIFU exposure and **(B)** during the exposure. **(C)** Pressure modulated acoustic waveforms at the HIFU focus used in the PSH approach. PSH, pressure-modulated shockwave histotripsy.

In the present work, *in vivo* experiments on male Sprague-Dawley (SD) rats (6 to 8 week old and weighing 200 to 250 g) obtained from Koatech (Pyeongtaek, Republic of Korea) were carried out to study the feasibility of using PSH for (a) mechanically fractionating liver tissue as well as for (b) varying the degree of mechanical damage. **Figure 1** shows a schematic diagram of the experimental setup used in the present study. All animals were housed in a temperature-controlled room (23°C) with a relative humidity of 50 ± 10% and alternate light/dark conditions. All the experiments were performed according to the approved guidelines and regulations provided by the Ethics Committee and the Institutional Animal Care and Use Committee of Korea University College of Medicine. Prior to PSH exposure, the rat’s left lateral liver lobe was partially exteriorised to simplify the guidance of the HIFU focus (20, 23, 29). A 2.0 MHz single element HIFU transducer (aperture size: 64 mm, radius of curvature: 63.2 mm, H148, Sonic Concepts, USA) coupled with a custom-built holder filled with degassed and deionised water was then placed on the exteriorised liver and the field coupled through a 25-μm thin acoustically transparent film (Mylar, Professional Plastics, CA, USA). The HIFU transducer was excited by a function generator (33600A, Agilent, USA) and a power amplifier (1040L, ENI, USA). The axial and lateral full-width half maximum pressure dimensions of the HIFU transducer were 7.25 mm and 0.89 mm respectively, and the HIFU focus was 3 mm below the surface of the exteriorised liver. The transducer holder was attached to a three axis-manual positioning system (Dovetail XYZ stage, ST1, Seongnam, Republic of Korea). During the experiments, the peak positive (*P*_,1+_ and *P*_,2+_) and negative pressure values (*P*_1,–_ and *P*_2,–_) at the HIFU focus, pressure modulation time point (*t_m_*), pulse repetition frequency (PRF) and duty cycle (DC) were kept constant whilst the pulse length (*P_L_*) and the number of pulses were varied as listed in **Table 1**. For comparison, the same exposure condition but no pressure modulation (e.g., BH) was also employed to create a number of lesions in the left lateral liver lobe. Three lesions were produced within the same liver lobe.

**Table 1:** Pressure-modulated shockwave histotripsy and boiling histotripsy exposure conditions used in the present study.

Exposure types	Frequency [MHz]	*P*_1,+_ and *P*_1,–_ [MPa]^*^	*P*_2,+_ and *P*_2,–_ [MPa]^*^	*t_m_* [ms]^*^	PRF [Hz]^*^	Duty cycle* [%]	Pulse length (*P_L_*) [ms]	Number of pulses
**PSH**	2	89.1, –14.6	29.9, –9.6	4	1	1	5, 7, 10, 14, 24, 34	1, 10, 20
**BH**	2	89.1, –14.6	–	–	1	1	7	1, 10

^*^These values were obtained from (32). PSH, Pressure-modulated shockwave histotripsy; BH, Boiling histotripsy.

### 2.2 Surgical procedure

After two weeks of acclimation, general anesthesia was performed with 3.5% isoflurane in a 2:1 N2O/O2 mixture. The mixture of gas was maintained in the anesthesia chamber via rat’s inhalation through a 2.5% nasal cone. Rats were placed on the warm pad and subjected to the midline xipho-pubic laparotomy, as previously described in (23). Both peritoneum and xiphoid were bent and pushed aside using silk surgical sutures to expose the left lateral lobe of the liver. Sterile gauze was placed between the liver and stomach to remove the blood and prevent potential gastrointestinal damage induced by PSH or BH exposure. 8 SD rats with 10 different exposure conditions were used in the present study.

### 2.3 Histology

Animals were sacrificed immediately after the PSH or BH exposure using the CO_2_ chamber. After the exposure, a dimple on the liver surface due to the formation of a lesion underneath the surface of the liver was observed. This was used for identifying the lateral position of the lesion. Treated rat liver tissue containing a PSH or BH lesion was then collected and placed in 4% paraformaldehyde (PFA, Biosesang) for histological examination. The tissues were fixed in 4% PFA for 48 h and after dehydration in 70% ethanol, embedded in a paraffin block. Following the standard protocols (36), the paraffin embedded liver tissue was then cut in 4.5 μm thickness and stained with haematoxylin and eosin (H&E) or Masson’s trichrome (MT). Haematoxylin stains cell nuclei as blue/purple colour whereas eosin stains cytoplasm and collagen as pink colour. With Masson’s trichrome staining, connective tissue (extracellular matrix, collagen) is stained blue, cytoplasm is stained red or pink, and nuclei are stained dark red or purple. Stained sections were imaged using the EVOS m7000 imaging system (Thermo Fisher Scientific) and Zeiss Axio Scan Z1 (Carl Zeiss).

### 2.4 Statistical analysis

In the present study, the size of a PSH or a BH lesion (i.e., no pressure modulation case) produced in the liver *in vivo* (axial length and lateral width) was measured from histological results. Statistical analysis on the lesion size difference was carried out at single 7-ms long PSH and BH pulses. Data were expressed as mean ± standard deviation. Distribution of normality was tested with the Shapiro–Wilk test and Student’s *t*-test was used for comparison. A *p*-value equal or less than 0.05 was considered statistically significant.

## 3 Results

### 3.1 Partial mechanical destruction of liver tissue by PSH

**Figure 2:**
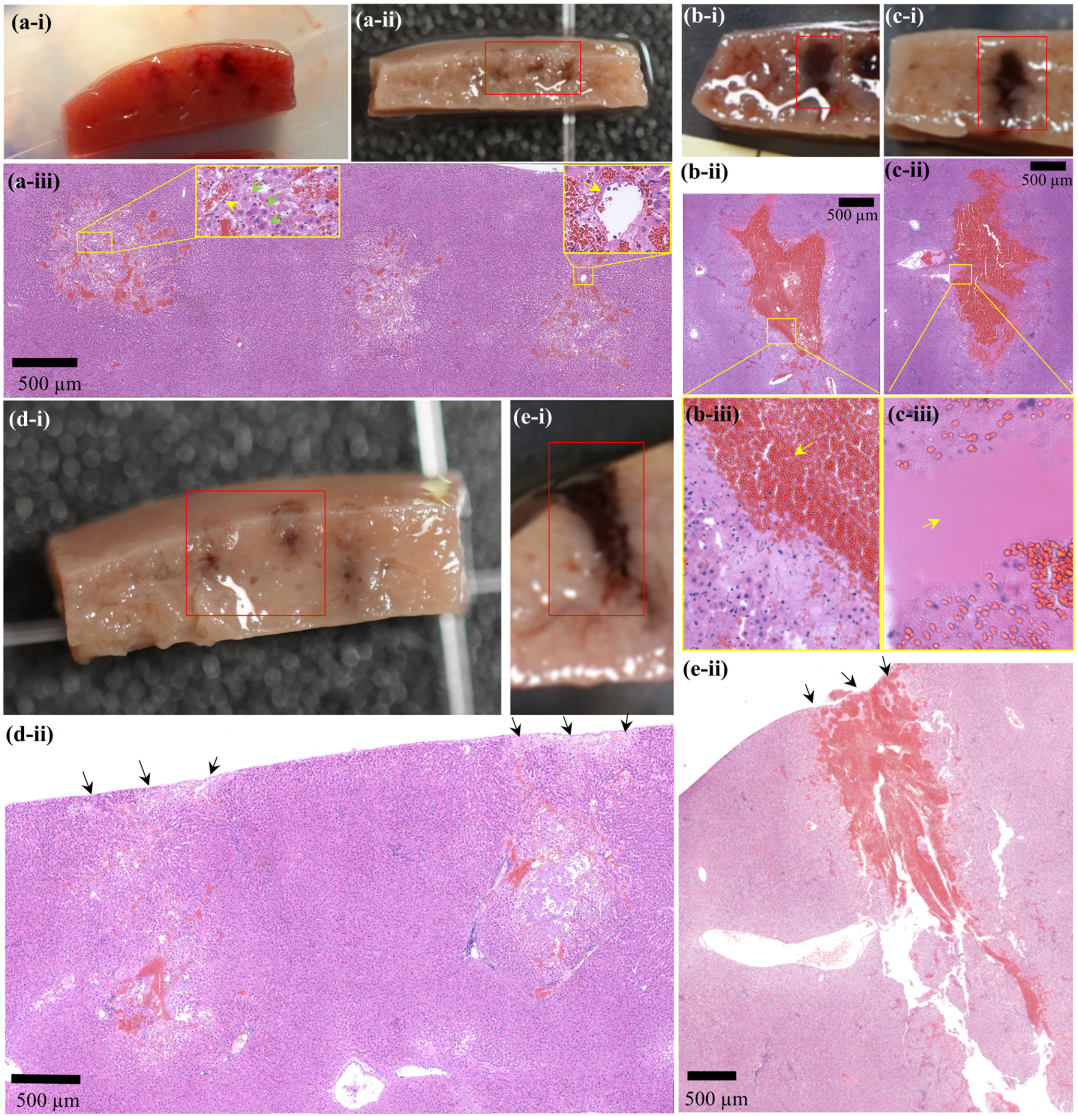
Morphological and histological observations of freshly created PSH or BH lesions in the liver *in vivo*. A cross-sectioned PSH lesion produced by **(A)** 1 pulse, **(B)** 10 pulses, and **(C)** 20 pulses with a pulse length of 7 ms, *P*_,1+_ of 89.1 MPa, *P*_,1–_ of – 14.6 MPa, *P*_,2+_ of 29.9 MPa, *P*_,2–_ of – 9.6 MPa, *t_m_* of 4 ms, 1 Hz PRF and 1% DC. **(Ai)** A photograph of three cross-sectioned PSH lesions after the exposure. **(Aii)** Formalin-preserved section of **(Ai)**. **(Aiii, Bii, Cii)** are the corresponding H&E stained liver tissues indicated in **(Aii, Bi, Ci)**, respectively. **(Biii, Ciii)** are higher magnification of the areas indicated by the yellow squares in **(Bii, Cii)** respectively. Formalin-preserved cross-sectioned BH lesions produced by **(Di)** 1 pulse and **(Ei)** 10 pulses with a pulse length of 7 ms, *P*_+_ of 89.1 MPa, *P*_–_ of – 14.6 MPa, 1 Hz PRF and 1% DC. **(Dii, Eii)** are the corresponding H&E stained liver tissues indicated in **(Di, Ei)**. The HIFU beam propagates from top to bottom.

To investigate the feasibility of using PSH for mechanically fractionating liver tissue, a partially exteriorised *in vivo* liver was initially exposed to various PSH exposure conditions. **Figure 2** shows morphological and histological observations of freshly generated PSH lesions in the *in vivo* rat liver. Five animals were used (one animal per a given pulse number). With a single 7 ms-long PSH pulse (*P*_,1+_ of 89.1 MPa, *P*_,1–_ of – 14.6 MPa, *P*_,2+_ of 29.9 MPa, *P*_,2–_ of – 9.6 MPa, *t_m_* of 4 ms, three lesions were produced), a partial fractionation in the shape of an oval with mean length of 1.04 mm ± 0.04 mm (mean ± standard deviation) and width of 0.87 ± 0.21 mm (mean ± standard deviation) can be observed (**Figures 2Ai–iii**). On macroscopic examination, no sign of coagulative necrosis induced by thermal damage is seen within and at the periphery of the PSH lesion. If thermal damage had appeared, more eosinophilic areas (i.e., stained a darker pink) with the occurrences of a shrunken hepatocytes nuclei and granular cytoplasm would have appeared (17, 37). Several preserved hepatic plates (indicated by the green arrows in **Figure 2Aiii**) and intact portal veins (indicated by the yellow arrows in **Figure 2Aiii**) within the PSH lesion can also be observed. The size of a PSH lesion enlarges and more severe mechanical destruction appears (i.e., higher degree of mechanical damage) with increasing the number of PSH pulses (10 and 20 pulses, **Figures 2B, C**). The length and width of a PSH lesion produced by 10 pulses are measured to be 2.93 mm and 1.17 mm (two lesions were produced, **Figures 2Bi,ii**) with the presence of coagulated blood in the lesion (indicated by the arrow in **Figure 2Biii**). With 20 PSH pulses, a larger lesion with a length of 3.43 mm and a width of 1.83 mm (two lesions were produced, **Figures 2Ci,ii**) is generated and is partially filled with homogenised liver tissue (indicated by the arrow in **Figure 2Ciii**). No normal hepatocytes are observed within this lesion. For comparison, BH is also used to produce a number of lesions in the liver, which are shown in **Figures 2D, E**. The mean length and width of a BH lesion produced with a single 7 ms-long BH pulse (*P*_+_ of 89.1 MPa, *P*_–_ of – 14.6 MPa) are measured to be 2.47 mm ± 0.72 mm (mean ± standard deviation) and 1.17 ± 0.19 mm (mean ± standard deviation) (three lesions were produced, **Figures 2Di,ii**), and a larger BH lesion (length of 4.58 mm and width of 1.25 mm) with the shape of a tadpole-like forms under 10 BH pulses (two lesions were produced, **Figures 2Ei,ii**). The width of the head of the BH lesion is 1.25 mm and that of the tail part is 0.5 mm (**Figure 2Eii**). In contrast to the extent of the PSH lesions shown in **Figures 2A, B**, there is a mechanical damage occurring on the surface of the liver after the BH exposure which is indicated by the black arrows in **Figures 2Dii**, **Eii**. This surface damage is most likely to be due to the presence of the shock scattering effect during the BH insonation (29–31). The comparison of a lesion size between PSH and BH is summarised in **Table 2**.

**Table 2:** Measurement of the size of a lesion produced in the liver *in vivo* by pressure-modulated shockwave histotripsy (PSH) and boiling histotripsy (BH).

	Pressure-modulated shockwave histotripsy		Boiling histotripsy	
Number of pulses	Length [mm]	Width [mm]	Length [mm]	Width [mm]
1	1.04 ± 0.04*	0.87 ± 0.21*	2.47 ± 0.72*	1.17 ± 0.19*
10	2.93	1.17	4.58	1.25
20	3.43	1.83	–	–

*mean ± standard deviation.

The following exposure conditions were used for PSH: *P*_,1+_ of 89.1 MPa, *P*_,1–_ of – 14.6 MPa, *P*_,2+_ of 29.9 MPa, *P*_,2–_ of – 9.6 MPa, *P_L_* of 7 ms, *t_m_* of 4 ms, PRF of 1 Hz and DC of 1%, and for BH: *P_+_* of 89.1 MPa, *P_–_* of – 14.6 MPa, *P_L_* of 7 ms, PRF of 1 Hz and DC of 1%.

### 3.2 Variation of a degree of mechanical damage induced by PSH

**Figure 3:**
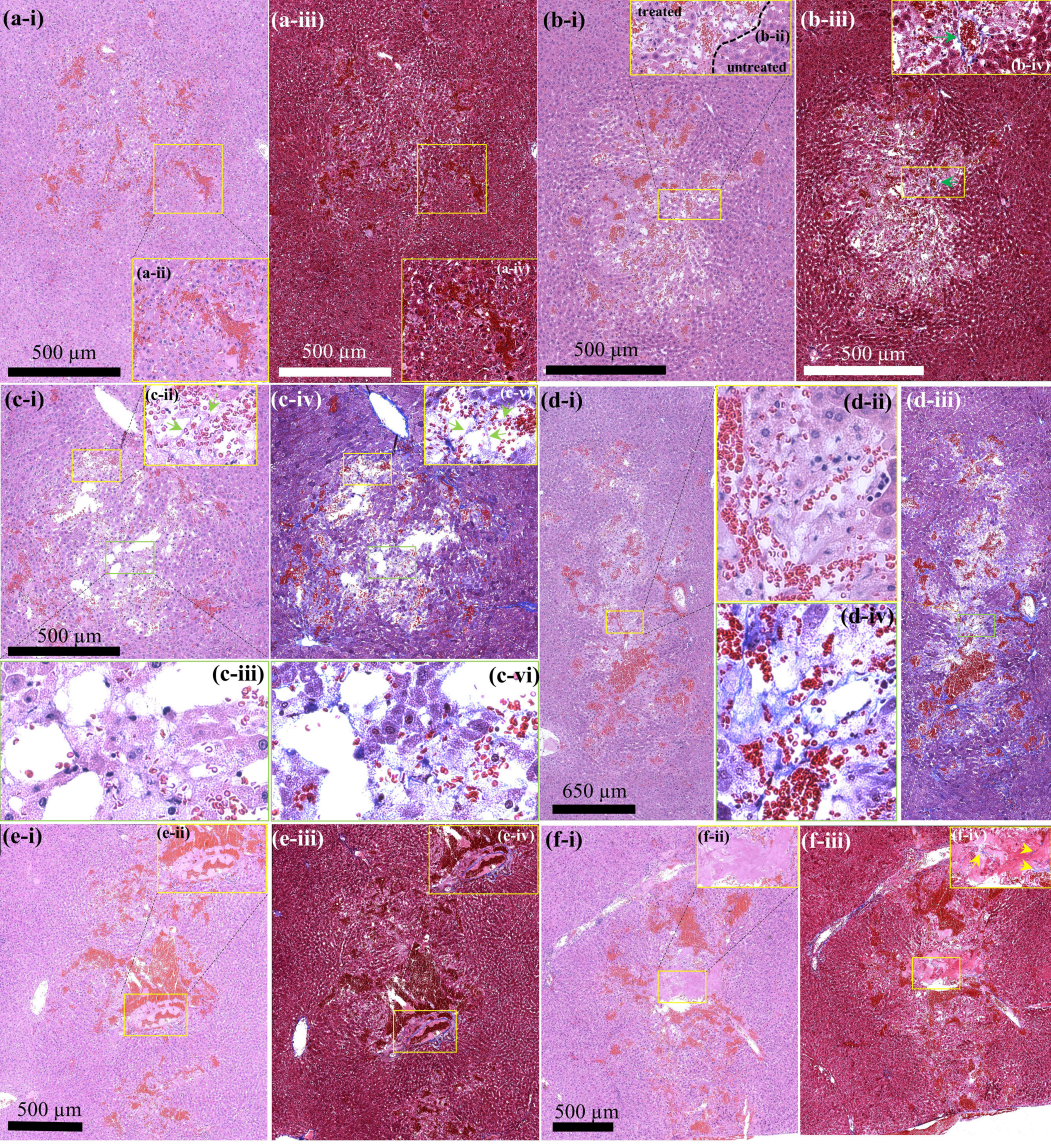
Histological examination of PSH lesions produced in the liver *in vivo* by a single PSH pulse with *P*_,1+_ of 89.1 MPa, *P*_,1–_ of – 14.6 MPa, *P*_,2+_ of 29.9 MPa, *P*_,2–_ of – 9.6 MPa at HIFU focus and *t_m_* of 4 ms. H&E stained cross-sectioned PSH lesion induced by **(Ai)** a 5-ms long PSH pulse, **(Bi)** a 7-ms long PSH pulse, **(Ci)** a 10-ms long PSH pulse, **(Di)** a 14-ms long PSH pulse, **(Ei)** a 24-ms long PSH pulse, and **(Fi)** 34-ms long PSH pulse. Corresponding MT stained cross-sectioned PSH lesions of **(A–F, i)** are shown in **(Aiii, Biii, Civ, Diii, Eiii, f-iii)** respectively. **(Aii,iv, Bii,iv, Cii,iii,v,vi, Dii,iv, Eii,iv, Fii,iv)** are higher magnifications of the areas indicated by the squares in **(Ai, Aiii, Bi, Biii, Ci, Civ, Di, Diii, Ei, Eiii, Fi, Fiii)**, respectively. The HIFU beam propagates from top to bottom.

An additional set of experiments was performed to investigate the feasibility of employing PSH for varying the degree of mechanical damage of liver tissue at the HIFU focus. A single PSH pulse was used whilst changing the pulse length (*P_L_*) from 5 to 34 ms. The same peak pressure magnitudes at the HIFU focus (*P*_,1+_ = 89.1 MPa, *P*_,1–_ = – 14.6 MPa and *P*_,2+_ = 29.9 MPa, *P*_,2–_ = – 9.6 MPa) and the pressure modulation time point (*t_m_* at 4 ms) used to obtain **Figures 2A, B** were employed. During the experiments, two lesions were produced at a given PSH pulse length. **Figure 3** depicts histological observations of freshly created PSH lesions at a given exposure condition (i.e., *P_L_* at 5, 7, 10, 14, 24 or 34 ms with *t_m_* at 4 ms). It can be observed that the degree of mechanical damage of liver tissue increases with increasing the pulse length. Throughout the various exposure conditions tested in the experiments, the lowest degree of partial destruction occurs with the shortest PSH pulse length (i.e., *P_L_* of 5 ms) where the structure of hepatic lobules is well preserved along with intact blood vessels and bile ducts within the lesion (**Figures 3Ai–iv**). With *P_L_* of 7 ms, hepatic plates can still be seen within the treated region; however, its structure is less compact (**Figures 3Bi–iii**) compared to that produced by *P_L_* at 5 ms (**Figure 3Aii**). Nevertheless, portal veins are well preserved within the treated zone, which is confirmed by the Masson’s trichrome (MT) staining analysis, as shown in **Figures 3Biii, iv** (indicated by the green arrows). By further increasing *P_L_* (i.e., 10, 14, 24 and 34 ms), the degree of mechanical fractionation gradually increases (**Figures 3Ci, iv, Di, iii, Ei, iii, Fi, iii**) with the presence of homogenised liver tissue within the treated regions (**Figure 3Fi, ii**). In addition to this, the axial (i.e., length) and lateral (i.e., width) dimensions of the PSH lesion also enlarge with *P_L_*; however, the lateral dimension does not change as much as the axial direction. This is summarised in **Table 3**.

**Table 3:** Axial and lateral dimensions of the PSH lesion produced in the liver at a given exposure condition.

Pulse length *P_L_* [ms]	Axial dimension (length) of the lesion [mm]	Lateral dimension (width) of the lesion [mm]
5	0.88	0.61
7	0.98	0.53
10	0.86	0.88
14	2.51	0.96
24	2.02	0.92
34	2.50	1.02

A single PSH pulse was used with the pressure modulation time point at *t_m_* = 4 ms. The axial and lateral dimensions were measured from **Figure 3**.

**Figure 4:**
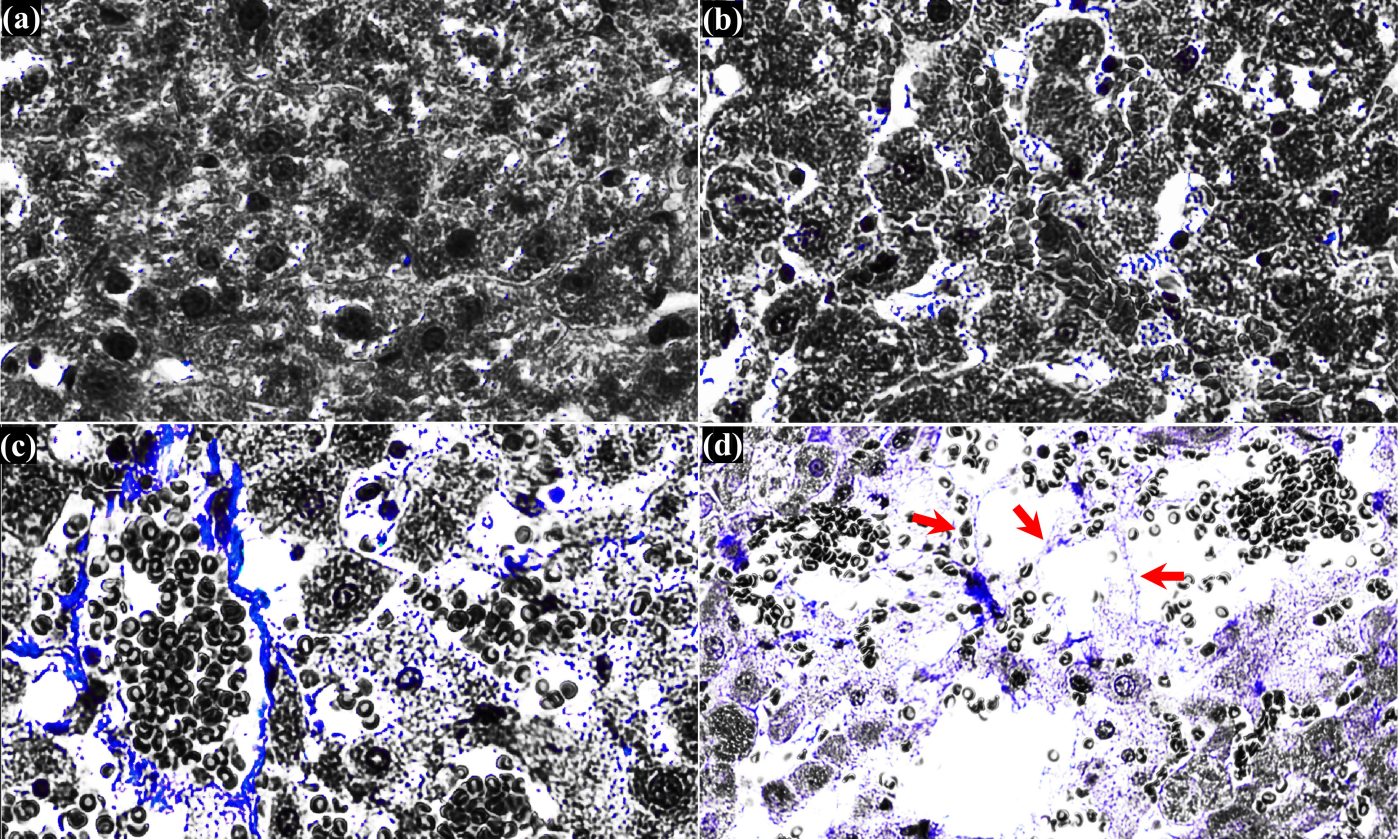
Histological observation of cross-sectioned MT-stained treated liver tissue. **(A)** untreated liver tissue. Treated liver tissue with a single **(B)** 5 ms long PSH pulse, **(C)** 7 ms long PSH pulse and **(D)** 10 ms long PSH pulse. Red arrows indicate the decellularised liver scaffold. The raw MT-stained image of **(D)** is obtained from **Figure 3Cv**.

At higher magnification (**Figures 3Cii, iii, v, vi, Dii, iv, Eii, iv, Fii, iv**), it can be noticed that fewer hepatic lobules are observed with some nuclear fragments of hepatocytes in the treated region at *P_L_* of 10 ms (**Figures 3Cii, iii**) compared to those observed under the shorter *P_L_* (5 ms and 7 ms, shown in **Figures 3Aii, iv, Bii, iv**). Interestingly, corresponding MT-stained section of **Figure 3Ci** reveals intact fibrillar collagen-like structure (indicated by the green arrows in **Figure 3Cv**), possibly indicating decellularised region where liver tissue is partially destructed with intact extracellular matrix and vascular network (20). Intact fibrillar collagen can also be seen within the homogenised region in the liver, as shown in **Figure 3Fiv** (indicated by the yellow arrows). Raw images of MT-stained treated liver sections were also filtered by colour in order to emphasise and identify the presence of fibrillar collagen. Since collagen is stained blue with MT staining, all colours of the raw images were filtered out except blue using a commercial software (Adobe Photoshop Ps, Adobe Inc, CA, USA), which are shown in **Figure 4**. In comparison to untreated liver tissue (**Figure 4A**), more blue coloured regions (i.e., fibrillar collagen structure) occur with increasing *P_L_* as shown in **Figures 4B, C**. Decellularised liver scaffolds can be observed at *P_L_* = 10 ms which are indicated by the red arrows in **Figure 4D**.

## 4 Discussion

For treating liver tumours adjacent to other organs, normal liver tissue, major vasculatures and/or bile ducts, precise mechanical destruction by BH is essentially needed. Whilst it has been found that there is a positive relationship between the extent of a BH lesion with the number of BH pulses (13, 17, 28), it is still challenging to precisely control its size because of the appearance of stochastic shock scattering effect in each BH pulse which primarily depends upon the size and location of a boiling bubble within the HIFU focal volume, and the sum of the incident field and that scattered by a bubble (32). For inducing more precise tissue damage than BH, a new histotripsy termed pressure-modulated shockwave histotripsy (PSH), where a pressure modulated shockwave is employed to produce, coalescence and maintain boiling vapour bubbles at the HIFU focus whilst eliminating the shock scattering effect, has recently been proposed by Pahk in 2021 (32) as an alternative to or in addition to BH. In a PSH pulse, the pressure modulation time point at which the peak pressure amplitudes reduce from *P*_1,+_ and *P*_1,-_ to *P*_2,+_ and *P*_2,-_ takes place at the time to reach boiling temperature (i.e., time-to-boil) which can be numerically predicted by solving the bioheat transfer equation or using the weak shock theory (13, 28, 29, 32). In the present study, for the first time, the effects of PSH on *in vivo* liver tissue were investigated morphologically and histologically with varying PSH exposure conditions, particularly the number of PSH pulses (1 to 20 pulses) and the length of a single PSH pulse (5 to 34 ms). The experimental results shown in **Figure 2** and **Table 2** demonstrated that PSH produced 2.37 times in length (*p* = 0.027) and 1.35 times in width smaller lesion (*p* = 0.1295) than that generated by BH in the liver under the same number of pulse (i.e., 1 pulse) and pulse length (i.e., 7 ms). The greater reduction in the axial direction (i.e., length) than that in the lateral direction (i.e., width) of the PSH lesion (**Figures 2Dii, iii**) is likely because that the shock scattering effect predominantly occurs along the axial direction towards the HIFU transducer in BH process (30). This is also the reason why there was no statistically significant difference between the width of a PSH lesion and that of a BH lesion produced under a 7-ms long single pulse. A significant difference would likely be observed with higher number of pulses and/or longer pulse duration. With PSH, backscattered peak negative pressure amplitudes by a boiling bubble can be kept below the cavitation cloud intrinsic threshold of – 28 MPa (15, 32). Similar to the changes of the size of a BH lesion with the number of BH pulses (**Figures 2D, E**), the size of a PSH lesion also enlarged with increasing the number of PSH pulses (**Figures 2A–C**). It can be speculated that the change of a PSH lesion dimension would be dependent upon the extent of a localised heated region occupied by boiling bubbles. As a shockwave heated zone broadens with an increase in the number of PSH pulses due to an accumulation of heat where the peak temperature does not return to ambient temperature between PSH pulses (13, 38), more enlarged boiling bubbles would form within this spatially localised heated region, resulting in the formation of an enlarged PSH lesion. However, as heat transfer processes reach equilibrium between PSH pulses, the tissue volume over which the shock wave heating appears also reaches a maximum (17), whereby the overall size of a PSH lesion would also reach saturation. The size of a PSH lesion also increases with the pulse length, as shown in **Figure 3** and **Table 3**. It can be noticed that the length of the PSH lesion gradually grew towards the opposite direction to the HIFU transducer along the axial direction with the pulse length, leading to the generation of an elongated lesion in the liver (**Figures 3D–F**). This increase in the axial direction is most probably due to the translational bubble movement by the HIFU radiation force during PSH exposure, which was observed in Pahk (32). This bubble movement can possibly be minimised by using a lower pressure amplitude of *P*_2,+_ than used in the present study as the radiation force is proportional to acoustic pressure amplitude. Besides the difference in the size of a lesion produced between PSH and BH, the shape of a PSH lesion is also different to that of a BH lesion. With the PSH exposure conditions used in the present study, an oval-shaped lesion was produced (**Figures 2A–C)** as compared to a tadpole shape (i.e., head and tail, as shown in **Figure 2Eii**) which is a typical lesion shape induced by BH. In BH, pre-focal cavitation clouds appear to form the head part of a BH lesion, whereas this event is negligible in PSH process, thus resulting in an oval shape lesion through shear forces produced by boiling bubbles at the HIFU focus. These experimental observations on the lesion shape are consistent with the previous study of bubble dynamics under BH and PSH exposures in an optically transparent liver tissue phantom with a high-speed camera (32). For effectively treating large solid tumours, PSH could be used together with BH. For instance, BH can initially be applied to mechanically fractionate a large area of solid tumours and then PSH could be employed to destruct the remnant tumours closely adjacent to normal healthy tissue, major blood vessels or other organs.

In the present study, the effects of changes of a PSH pulse length on the degree of mechanical damage induced in the left lateral liver lobe were also examined. Interestingly, the damage degree of liver tissue gradually increased with increasing the pulse length (**Figures 3**, **4**). In all the experiments, portal veins and bile ducts were intact within the PSH lesions in the liver. With the shortest pulse length, most hepatic lobules including hepatic sinusoids were well preserved with minor liver damage (**Figures 3Ai, iii**). These hepatic structures became loosen with a slightly longer pulse length (**Figures 3A–F**), and decellularised liver scaffolds eventually appeared (**Figures 3Cii–iv, vi**, **4D**). By further increased the pulse length beyond 10 ms at which the liver tissue decellularisation was observed, a completely homogenised region was found within the PSH lesion (**Figure 3Fii**). These experimental results can reveal the tissue selectivity of PSH. This tissue sparing or partial decellularisation effect is likely to be due to the differences in the mechanical properties of different types of tissue, leading to a variation in the susceptibility to mechanical damage (39). This is of paramount importance for cell therapy as an *in situ* decellularised lesion with intact extracellular matrix and vascular network could (a) promote cell attachment and proliferation and (b) supply sufficient oxygen and nutrients to transplanted cells to survive (14, 20, 29, 40, 41). For liver cancer treatment, normal healthy hepatocytes could be injected or transplanted directly into a PSH-induced decellularised region where liver cancer cells were selectively fractionated for remodelling tumour microenvironment. In fact, it has been demonstrated that BH can be employed to result in partial tissue decellularisation for cell therapy (20) and regenerative medicine (40) as connective tissue structures in the liver such as hepatic blood vessels and bile ducts are more resistant to BH treatment than cellular tissue due to the higher tensile strength of collagen (16, 20, 40, 41). Pahk et al. (29) numerically showed that strain induced by the formation and dynamics of a boiling bubble under BH exposure in the liver is above the ultimate fractional strain of liver but is below that of femoral artery. This numerical result indicates the importance of controlling the dynamics of boiling bubbles on decellularisation. From this perspective, since (a) shear stresses generated by boiling bubble(s) in the absence of the shock scattering effect (32) are the main cause of mechanical tissue destruction in a PSH process (**Figures 2**, **3**) and (b) the lifespan of a boiling bubble (32) and (c) the degree of damage can be controlled through changing a PSH pulse length (**Figures 3**, **4**), PSH could potentially provide higher degrees of tissue selectivity and treatment accuracy than BH. Though a long-term follow up study including the investigation of immune response to PSH was not performed in the present study, similar immunological effects induced by a conventional histotripsy approach would be observed by PSH because PSH is a type of histotripsy technique (i.e., mechanical destruction of solid tumours via acoustic cavitation at the focus). Different PSH exposure conditions would vary the extent and degree of mechanical damage; thereby possibly affecting the level of immune response. Furthermore, since acoustic properties of liver cancer tissue are different to normal liver tissue (cancer tissue is stiffer), the size as well as the shape of a PSH lesion produced in solid liver tumours may possibly be different to the results shown in the present study. This warrants further investigations including optimisation of PSH exposure conditions.

Pressure magnitudes of a pressure modulated shockwave pulse (*P*_,1+_, *P*_,1–_ and *P*_,2+_ and *P*_,2–_) and pressure modulation time point (*t_m_*) are the two essential parameters involved in PSH method. In the present study, the PSH exposure conditions used in the experiments were adopted from Pahk (32). The tissue phantom used in Pahk (32) has similar acoustic and thermal properties to those of liver except for the attenuation coefficient which is 0.15 dB cm^-1^ MHz^-1^, rather than that for liver which is 0.52 dB cm^-1^ MHz^-1^ (13, 42). This difference in the attenuation coefficient may slightly shift the time-to-boil in the *in vivo* liver earlier than that in the liver tissue phantom which was predicted to be 3.98 ms (32). Therefore, further smaller PSH lesions than the smallest lesion size observed in the present study (i.e., 0.88 mm in length and 0.61 mm in width, shown in **Figure 3Ai** and **Table 3**) could be generated with the use of a shorter *t_m_* than 4 ms.

In conclusion, the experimental results presented in this study together with Pahk (32) can suggest that pressure-modulated shockwave histotripsy could be an invaluable tool as (a) precise tissue fractionation method for inducing highly localised liver tumour destruction and immune response, as well as (b) cell selective destruction method for cell therapy or regenerative medicine in the future. Further work will be carried out to investigate potential immunological benefits and adverse effects of PSH for cancer treatment with the exposure conditions used in the present study.

## Data availability statement

The original contributions presented in the study are included in the article/supplementary material. Further inquiries can be directed to the corresponding author.

## Ethics statement

All the experiments were performed according to the approved guidelines and regulations provided by the Ethics Committee and the Institutional Animal Care and Use Committee of Korea University College of Medicine.

## Author contributions

KJP conception and design of the work, acquisition, analysis and interpretation of data, drafting and revising of the article, and study supervision. JH and CJ design of the work, acquisition and analysis of data, histology. KP design of the work, acquisition, analysis and interpretation of data. All authors contributed to the article and approved the submitted version.
